# Motor errors lead to enhanced performance in older adults

**DOI:** 10.1038/s41598-017-03430-4

**Published:** 2017-06-12

**Authors:** S. Levy-Tzedek

**Affiliations:** 1 Recanati School for Community Health Professions, Department of Physical Therapy, Ben-Gurion University of the Negev, Beer-Sheva, Israel; 2 Zlotowski Center for Neuroscience, Ben-Gurion University of the Negev, Beer-Sheva, Israel

## Abstract

Young individuals make larger and faster forearm movements when visual feedback about the movement is not available, compared to when it is. We set out to test whether this behavior persists with aging. We tested 40 participants, 20 in each age group – young and old, on a task that required making rhythmic movements of the forearm with and without visual feedback. Surprisingly, we found that older adults increased the speed and the amplitude of their movements to an even greater extent than did the young adults. Furthermore, we found that the increase in speed and amplitude during the non-vision trial segments improved their performance on the task, and they were able to leverage the change in these movement parameters (speed and amplitude) to improve their performance during subsequent trial segments that did include visual feedback. The improvement in accuracy on the task was accompanied by a decrease in path variability. The results indicate that older adults can adapt their movement parameters to enhance performance following a motor perturbation. They further suggest that motor variability in old age can be advantageous under certain circumstances.

## Introduction

When young adults make rhythmic movements of the forearm, the movements are larger and faster in the absence of visual feedback, compared to when visual feedback is available^1^. This is not unique to the forearm - larger-amplitude movements were also found when visual feedback was not available for movements of the wrist^2^. This is a curious finding, which may have several explanations. One explanation is that this behavior is the result of a mismatch between the perception and the actual generation of amplitude^3, 4^ and speed, possibly reflecting a degraded performance of proprioception compared to vision^5^. Shergill *et al*.^6^ found a parallel pattern in force generation. According to this explanation, when visual feedback is not available, the movements are perceived via proprioception as smaller and slower than they actually are, and the evident increase in size and speed is a result of compensating for the misperception of size and speed. Another possible explanation is that in the absence of visual feedback, a stronger proprioceptive signal is needed in order to assert the location of the limb in space (for related evidence in locust flight, see ref. 7), and the larger and faster movements are aimed at generating a stronger signal^8^.

A series of experiments in young adults demonstrated that this is not a transient behavior, but rather a consistent one, which does not diminish when one becomes aware of this unwarranted increase in both speed and amplitude of movement^1^.

Healthy older adults, as well in individuals with Parkinson’s disease, also show an increase in amplitude and speed of their forearm movements in the absence of visual feedback^9^. But would this increase in amplitude and speed persist over time and despite awareness, as it does in young adults?

Older adults are known to suffer more from fatigue, compared to young adults^10, 11^. Their movements are often smaller^12^ and slower^13, 14^ than those of younger individuals. These findings led us to ask two separate but related questions: We asked whether the older population would, as the young did, maintain a larger and faster movement pattern in the absence of visual feedback, over time, despite awareness – or would this pattern diminish over repetitions. We further asked whether, if the increased speed and amplitude when no visual feedback is available led the older adults to enhanced performance on the task, they would be able to learn from this so-called “motor error”, and use this benefit on subsequent visually controlled motor tasks (or in other words, would they successfully adapt their movement pattern following a motor error).

We hypothesized that they would not maintain an increase in speed and amplitude of movement over time due to fatigue, and that they would not adapt their movement pattern during visual trials, even if the increase in speed and amplitude led to improved behavior.

## Methods

### Equipment

**Figure 1:**
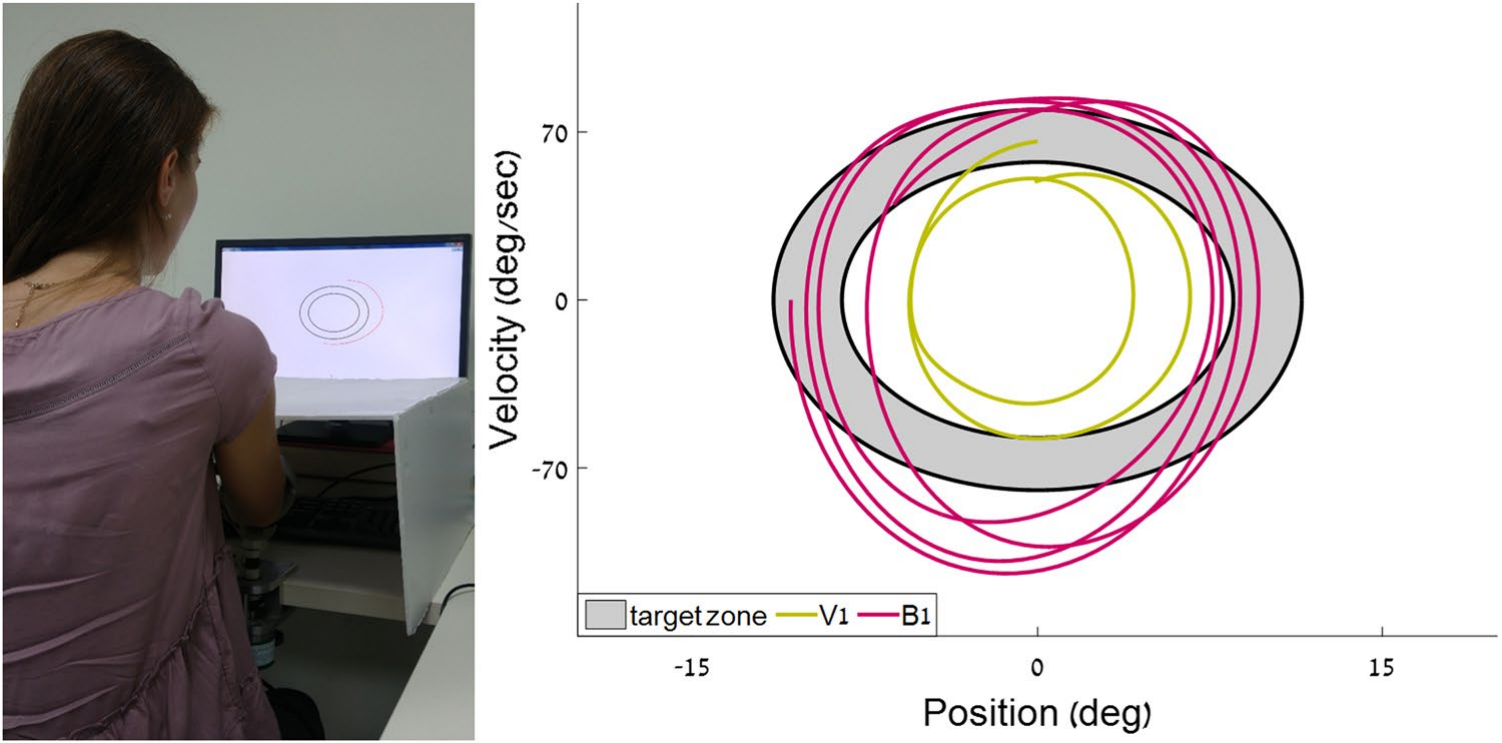
*Left:* The experimental setup. The participant places her forearm on the armrest, below an opaque cover. She uses the movements of her forearm to control a cursor on a phase plane, displayed on a computer screen. Shown here is the moment of transition between the first blind segment (B1), and the second visual segment (V2), when the red trace of her movement just reappears on the screen, and is outside the prescribed limits of the movement imposed by the black ellipses (see also supplementary video S1). *Right:* an excerpt from the movement traces of an older participant in the current experiment. The gray zone delimited by two black lines is the target zone on the phase plane, within which participants are asked to keep the cursor (during the actual experiment, the area between the two black ellipses was white – shown here in gray for clarity). The olive line shows the movement trace during a portion of this participant’s V1 segment, and the magenta line shows the movement trace during a portion of this participant’s B1 segment. It demonstrates the increase in both speed and amplitude during the blind segments, compared to the visually guided ones.

**Figure 2:**
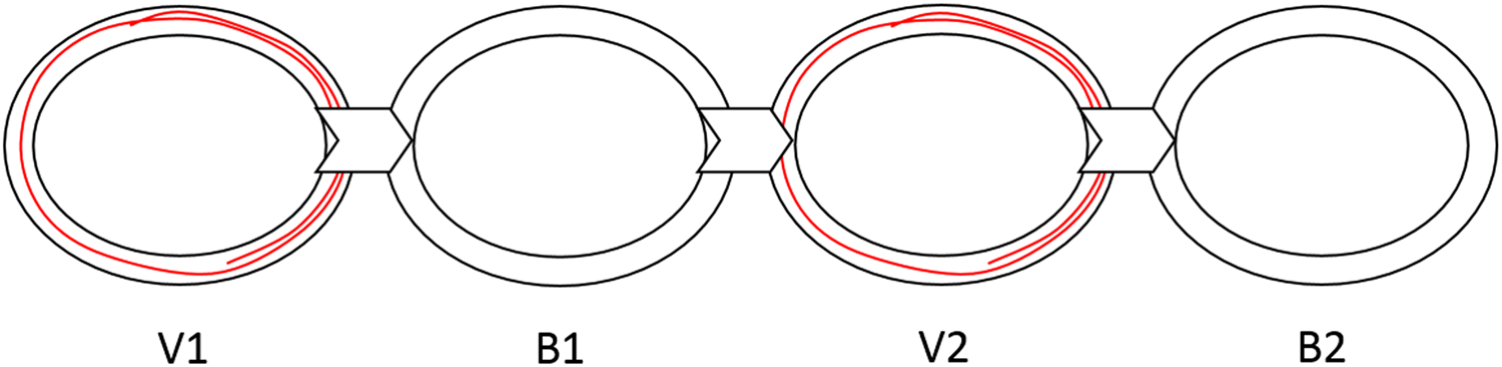
An illustration of the four segments of each trial. Two concentric black ellipses mark the target area on the phase plane, within which participants should maintain the cursor. During V1 and V2 (each lasting 10 sec), a red trace representing the forearm movement is displayed on the screen. During B1 and B2 (each lasting 20 sec), the trace of the movement is not displayed on the screen.

We used the experimental apparatus described in refs 1, 9, 15–18. It comprises an arm rest, connected to an encoder that records the rotation of the arm about the elbow with an accuracy of 0.002 degrees at 200 Hz. The arm rest was designed to be as light as possible, to minimize its effect on the natural movement of the arm. The arm rest was free to rotate around its axis, with no imposed limits on the range of motion. Participants placed their arm on the arm rest, situated parallel to the table on which it was mounted, and moved their forearm towards and away from their body in a movement similar to that of a windshield wiper. An opaque cover was placed on the table, above the experimental apparatus, such that no direct visual information about the arm’s position was available. Participants received real-time feedback on their arm’s location and speed via a cursor that appeared on the computer screen placed on the table in front of them (see Fig. 1, left panel, and supplementary video S1). The cursor denoting the amplitude and the speed of the movement was available only during parts of each trial (the “visual” segments), and not during others (the “blind” segments; see Protocol below, and Fig. 2).

Participants’ arm was placed in a wrist brace, to prevent movements of the wrist. The forearm flexion/extension movements were one-dimensional, in the horizontal plane. No explicit timing cues were given.

### Protocol

The computer screen displayed a phase plane: position was displayed on the horizontal axis, and velocity on the vertical axis (see Fig. 1, right panel). On that phase plane, a pair of concentric ellipses created an enclosed doughnut-shaped area, that defined the lower and the upper bounds of the amplitude (on the X axis) and speed (on the Y axis) allowed on the task (marked in gray in Fig. 1, right panel). The participants controlled a cursor on the phase plane with their arm movements. The speed of their movements was represented by the height of the cursor, and the displacement of their movement was represented by the horizontal location of the cursor. They were asked to maintain the cursor within the doughnut-shaped area at all times. Thus, the amplitude, speed and frequency of the movement were prescribed.

#### Training

The training phase consisted of two parts. In part I, participants practiced controlling their arm movements such that the cursor on the phase plane stays within the prescribed ellipse on three different doughnut sizes on the phase plane. These practice ellipses had central frequencies of 0.1 Hz, 0.7 Hz and 2 Hz. The participants were given the option to repeat these practice trials, each lasting 40 seconds, until they felt comfortable with the task. The order at which these practice ellipses was presented across participants was counterbalanced. In part II, they were asked to maintain the cursor between the two ellipses, as the height of the ellipses was gradually increasing (requiring higher movement speed) or decreasing (requiring lower movement speed; see ref. 18). This enabled the participants the opportunity to experience movement at a range of speeds which is broader than the one required during the testing phase.

#### Testing

In the testing phase, there was a single doughnut size throughout, with a central frequency of 1.6 Hz (range 1.2–2.1 Hz). There were four trials, each lasting 62 sec. During each trial, we alternated the availability of the on-screen visual feedback. Thus, each trial was divided into four segments: two segments with on-screen visual feedback, and two segments with no on-screen visual feedback (see Fig. 2). During the “visual feedback” (V) segments of the trial, the cursor denoting the speed and the position of the forearm on the phase plane was displayed on the screen, along with a trace of the movement, and during the “blind” segments (B), the cursor and the trace were not displayed on the screen, and participants had no visual representation of their arm movements (see ref. 1). Each visual segment (V1 and V2) lasted 10 sec, followed by a 20-sec blind segment (B1 and B2, respectively; See Fig. 2) (an additional second was added at the beginning of V1 and another at the end of B2, and these were not included in the analysis, to avoid edge-effects).

In those parts of the trials, when visual feedback was not available, the participants were asked to continue and perform exactly the same movement as they did when visual feedback was available. The trials were specifically designed to include transitions between V and B segments within each trial. Of particular importance is the transition between segment B1 and segment V2, as it enables participants to get a snapshot of their performance during the blind segment (see Fig. 1, left panel and supplementary video S1). That is, in the first seconds of segment V2, movement is still controlled in a feed-forward manner from the B1 segment, and participants receive a visual representation of the amplitude and the speed of their movements during the blind segment.

Figure 2 depicts the changes in visual feedback during each trial. The participants started each trial at a neutral position, from which they could comfortably flex and extend their forearm, and this neutral position defined the center of the concentric ellipses (i.e., it is the point of zero position and zero velocity, prior to initiation of the movement).

All ellipses defined a target arm amplitude of 20 ± 3°, which corresponded to a visual angle of ~6 degrees.

Ethical approval for this study was obtained from the Ethics Committee at the Ben Gurion University of the Negev. All experimental procedures were performed in accordance with this ethical approval.

### Participants

A total of 40 individuals participated in this experiment. 20 young adults (age: 24.4 ± 1.1 years old, mean ± SD; 13 females, 7 males) and 20 older adults (age: 70.5 ± 5.6 years; 16 females, 4 males) right-handed participants were tested using their right arm. All participants gave their written informed consent to participate, as stipulated by the Ben-Gurion University Committee on Ethics.

### Data analysis

The angular displacement of the forearm about the elbow joint was filtered and trend was removed from the position data to reduce the effects of drift. Data were analyzed with MATLAB® software (Mathworks, MA, v.8.5).

### Outcome measures

The following outcome measures were calculated separately for each of the four trial segments (V1, B1, V2, B2).

#### Phase-plane area

We calculated the area taken up on the phase plane by the movements of the participants, thereby capturing the concurrent changes in both amplitude and speed^1^. The reported trends in phase plane apply to amplitude and peak speed when analyzed separately as well.

#### Frequency

The average frequency of the rhythmic forearm movements was calculated by taking the inverse of the average time to complete a movement cycle.

#### Accuracy

Each trial segment was given a numerical score that represented the percent of the total trial time that was spent inside the target zone on the phase plane^16^ (marked in gray on Fig. 1, right panel).

#### Variability in phase-plane path

Movement traces from each segment in each trial were resampled to a uniform length. The edges of the resultant vectors were removed, to avoid edge-effects due to resampling. The values from each quadrant were then normalized by the maximum value in that segment, and the variability in the path was calculated by taking the standard deviation of the path across the repetitions within the given segment^16^.

**Figure 3:**
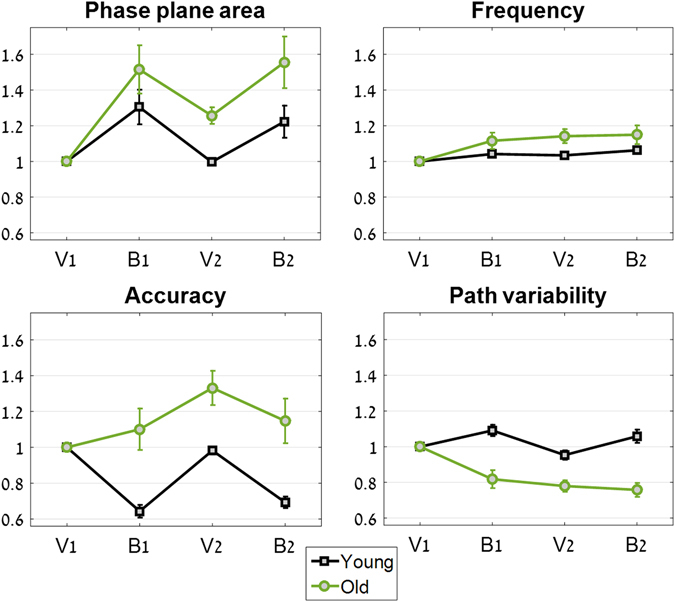
Performance by age groups across the four trial segments. For clarity, all segment values are normalized by the first segment of the trial (V1). The results of the young group are marked in black, and those of the older group are marked in green. Error bars represent standard error. (**A**) Normalized phase-plane area values; (**B**) normalized frequency values; (**C**) normalized accuracy scores; (**D**) normalized path variability.

For the sake of clarity, Fig. 3 shows the values of the outcome measures normalized by the values obtained in the first visually guided segment (V1)^1^ (the normalization was performed per individual). In this manner, it is possible to capture the overall relative changes in this outcome measure over the four trial segments, despite individual differences in initial values.

### Statistical analysis

To test the first hypothesis, that movement of the older participants during the blind segments would not be larger and faster than movements during the visual segments, we used a two-sided Wilcoxon signed rank test, testing whether the difference in phase-plane area between the blind and the visual segments has a distribution whose median is different from zero. To test the second hypothesis, that there will be no difference in performance between the two visual segments, we used the Kruskal-Wallis test to compare the values obtained during the two segments of interest - V1 and V2, for each outcome measure. The non-normalized values were used for the statistical analysis. Each age group was analyzed separately. No direct comparison between the young and the old group was done, since this was not one of the study goals. Non-parametric tests were chosen to eliminate the need for assumptions regarding population distributions required in parametric tests.

### Data Availability

The datasets generated during the current study are available from the corresponding author on reasonable request.

## Results

### Phase plane area

The results from the young group replicate our previously reported performance in the young^1^. In the B1 segment, there was a 31% increase in the area taken up on the phase plane; in V2, the area taken up on the phase plane was the same as in V1, and in B2, there was once again an increase in the area on the phase plane (by 22%, see Fig. 3A). The difference in phase-plane area between the blind and the visual segments in the young group was found to be significantly different from zero (Z = 3.1, p = 0.002). There was no statistically significant difference among the two visually guided segments V1 and V2 (χ^2^_(1,20)_ = 0.02, p = 0.9). The older group’s results were surprisingly different. B1 saw an increase of 52% in the area taken up on the phase plane, and during V2 there was no return to baseline (as there was in the young group), but rather a 26% increase in area compared to V1. During B2, the area taken up on the phase plane was 56% greater than in V1. The difference in phase-plane area between the blind and the visual segments in the older group was found to be significantly different from zero (Z = 4.3, p < 0.0001). There was a statistically significant difference between the two visually guided segments for the older group (χ^2^_(1,20)_ = 4.3, p = 0.04).

### Frequency

Here, too, the results of the young group replicated our previous findings^1^, and there was no significant difference in frequency between the two visually guided segments. (χ^2^_(1,20)_ = 0.5, p = 0.5), with frequency values in B1, V2 and B2 being on average only 4%, 3% and 6% higher than in V1, respectively (see Fig. 3B). In the older group, frequency values increased by 12%, 14% and 15% in those segments, respectively, compared to V1. Here, too, there was no significant difference between V1 and V2 (χ^2^_(1,20)_ = 2.0, p = 0.2). That is, both age groups demonstrated no significant change in frequency between the two visually guided segments.

### Accuracy

For the young group, the graph of the accuracy scores was the mirror image of the phase-plane area graph: in B1 there was a 36% decrease in accuracy, in V2 a return to baseline performance, and in B2 a decrease of 31% in accuracy compared with V1. That is, the increase in area taken up on the phase plane was accompanied by a decrease in accuracy scores. There was no significant difference between the accuracy scores in segment V1 compared to V2 for the young group (χ^2^_(1,20)_ = 0.09, p = 0.8).

In the older group we found a different pattern: accuracy results were on average 10% *higher* during B1, and then further increased in V2 to 33% higher than V1, with B2 being 15% more accurate on average than V1 (see Fig. 3C). The increase in accuracy scores between V1 and V2 was significant for the older group (χ^2^_(1,20)_ = 4.1, p = 0.04).

That is, unlike the young group, which had the same accuracy results in V1 and in V2, the older group significantly improved their accuracy scores between V1 and V2.

**Figure 4:**
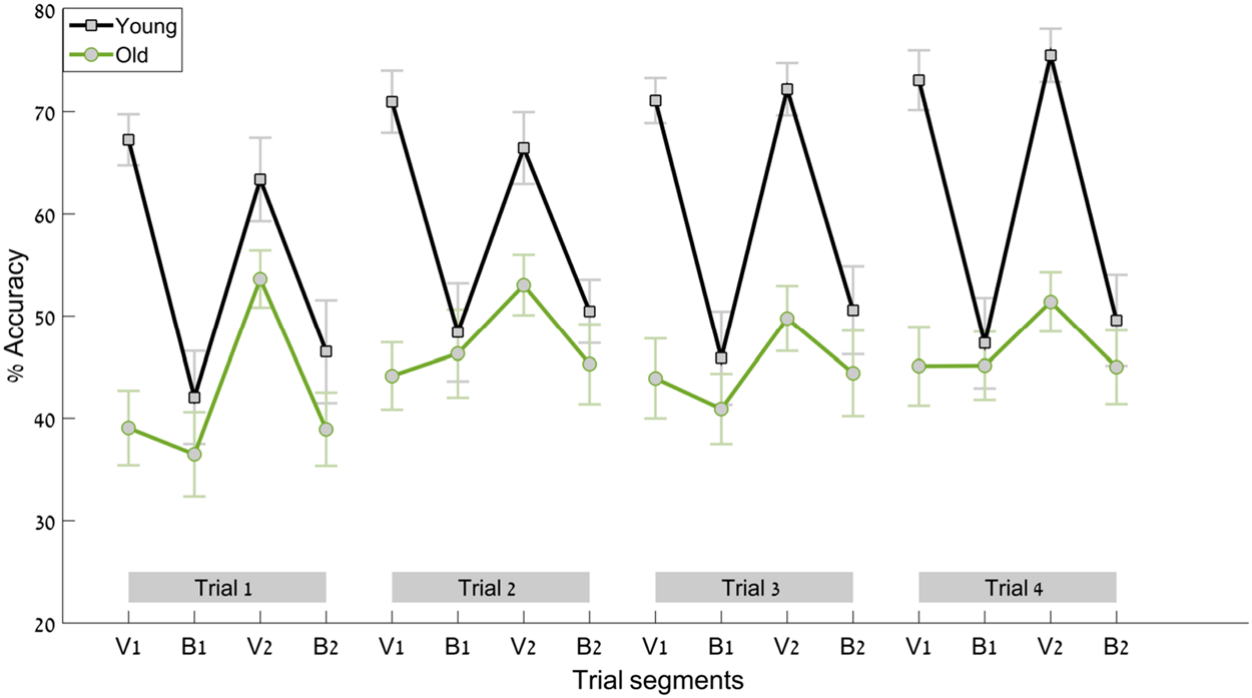
Accuracy. Shown here are the non-normalized accuracy values (the percent of the total trial time spent inside the target zone) for all 16 trial segments (four trials with four segments each), for the young group (in black) and for the old group (in green). Error bars denote standard error.

Figure 4 shows the actual (non-normalized) values for all 16 trial segments (4 trials times 4 segments per trial). It demonstrates that (i) there is no ceiling effect in the young group (that is, the reason we do not see similar behavior in the young group as in the old group is not because they were performing at 100% accuracy, as they did have room for improvement), and (ii) that older participant reached their highest performance value as early as V2 of the first trial, and did not improve beyond that during the experiment (and thus were not in a continuous learning phase throughout the experiment).

### Path variability

The variability in the movement path in the young group increased from V1 to B1 by 9%, decreased by 5% in V2 compared to V1, and rose by 6% in B2 compared to V1. The difference between V1 and V2 was not significant (χ^2^_(1,20)_ = 1.0, p = 0.3). In the old group, however, there was a continuous drop across the trial segments, with B1, V2 and B2 having 18%, 22% and 24% less path variability, respectively, with respect to V1 (see Fig. 3D). The reduction in path variability between V1 and V2 was significant (χ^2^_(1,20)_ = 11.6, p = 0.0007).

That is, while there was no change in path variability for the young group, the old group significantly reduced their path variability between segments V1 and V2.

#### Fatigue

At the end of the experiment, seven older adults and two young adults reported they experienced fatigue.

## Discussion

We had two goals in the current experiment: The first goal was to test whether older adults persist in making larger and faster movements in the absence of visual feedback, over multiple repetitions, and despite awareness of this increase, as do the young. We found that they do, as evidenced by the average 56% increase in area taken up on the phase plane during the second “blind” segment in each trial (B2).

Our second goal was to test whether the older adults were able to use this increase to benefit future movements. We found that they did. In transitioning from the first visually guided segment (V1) to the first segment with no visual feedback (B1), the older group experienced an increase in movement amplitude and speed. When visual feedback was restored (segment V2), they did not return to baseline behavior as did the young group. Rather, they were able to move at higher movement speed and amplitude than they initially did, in a manner that benefited task performance, and reached accuracy scores that were 33% better compared to the first visually guided segment (V1). In other words, the motor “error” in segment B1 served as a trigger for enhanced performance during the following visually guided segment (V2). We refer to this increase in amplitude and speed as a motor error since it is an *unintended* change in movement parameters, which has been assumed to be the result of misestimation of the performed movement parameters^1^. The term “motor error” may seem paradoxical since the accuracy scores, on average, were *higher* during the first “blind” segment (B1) than during the first visually guided segment (V1) for the older group (as seen in Fig. 3). However, this is not reflected in the average scores per trial (Fig. 4), and the evidence collected thus far^1, 9^ suggests that the motor changes that lead to this increase in accuracy were not intentional. Rather, the older participants increased the speed and the amplitude of their movements, as did the young participants, as the result of a motor error, such as a misestimation of the movement parameters. When the visual feedback was restored during V2, during the transition, both groups were able to see a glimpse of the trace they produced during B1. For the young group, this trace was often well outside the pre-defined bounds of the task, and they corrected their movement parameters, such that accuracy levels rose once again during V2. For the old group, this trace was on average closer to the task bounds than the trace they produced during V1. Rather than reverting to the movement parameters they used in V1, they maintained some of the increase in amplitude and speed from B1 to V2, as evident in the overall increase in phase-plane area from V1 to V2 (Fig. 3). That is, the unintentional increase in speed and in amplitude during the segment with no visual feedback served as a primer for improved performance when visual feedback was restored. This is a seemingly paradoxical situation in which a motor error leads to improved performance.

### Older adults show a greater increase in speed and amplitude in the absence of visual feedback

At first blush this seems to be a counterintuitive finding. Indeed, it contradicts our expectation that since older adults make smaller^12^ and slower^13, 14^ movements to begin with, and are more subject to the effects of fatigue^10, 11^, the increase in the speed and the amplitude of their movements will be less than that found for younger adults. However, this finding is very much in line with research showing a decrease in proprioceptive acuity with age^8, 14, 19, 20^, which suggests that the increase in speed and amplitude may be a way to enhance the incoming proprioceptive signal^8^. That is, the reduced proprioceptive acuity leads to larger and faster movement generation when no visual feedback is available, which, in turn, results in a larger movement error.

### The importance of making motor errors and variable movements

Making errors in control of movement plays a pivotal role in learning new actions, improving existing skills, and avoiding future mistakes^21^. Its role is highlighted in early development – primarily in infancy – when babies and toddlers explore the range of possible movements, from which they learn which movements are most successful in obtaining their goal (e.g. reaching for food or walking^22, 23^). Error monitoring of self^24^ and others^25, 26^ is an important part of successful control of movement, and is evident already in early childhood^27^. Adaptation of movement control as a result of monitoring of errors is done while considering the source of the error: if there is a transient, external source of error (such as a gust of wind derailing a tennis ball from its intended course), the motor system ignores it in future movement planning^28^. In the current experiment the motor error which occurred during the first non-vision segment of the trial was used to improve performance on the following visually guided segment. Bernstein has previously noted on how variability in movement is critical for finding an optimal solution for the performance of a movement^22^. Here, the space of possible variations on the target movement was expanded by the increase in amplitude and speed of movement that follows the elimination of visual feedback. During the non-vision segment, older participants found a more efficient movement pattern for attaining the goal of staying inside the target zone on the phase plane, and adapted their subsequent movements during the visually guided segment such that their accuracy levels were higher. Once they found a better set of movement parameters, they exhibited a concomitant significant decrease in movement variability (Fig. 3).

These results are similar to those found by Tyrell *et al*.^29^ in individuals who have had a stroke: at baseline, the participants showed an asymmetry in their gait pattern; during the experiment, the asymmetry (or “error”) was exaggerated using a split-belt treadmill manipulation, and when this perturbation was removed, the participants reached a stable, symmetric gait pattern which did not diminish for the duration of the test (5 mins). This was only the case when the error generated by the perturbation served to produce a *desired* symmetric gait pattern upon removal (in another condition, when a non-desired gait pattern emerged, the post-perturbation de-adaptation was quick). This, together with the current experimental results, are examples of how motor errors, whether self-generated, or externally imposed, can lead to enhanced performance, which is then maintained by the participants.

These results suggest that motor perturbations that lead to temporary “errors” can be beneficial, since the resultant motor adaptation^30, 31^ can be a desired end result (in the Tyrell *et al*. experiment it was a symmetrical gait pattern; in the current experiment is was a larger and faster movement pattern).

Paradoxically, old age is associated with both increased motor variability^10^ and reduced motor exploration^32^. Unlike findings in early development, which associate a positive outcome with motor variability, in older age, variability in motor output is often associated with reduced task performance (e.g. refs 23, 33). Here, we demonstrate that one form of motor variability – unintentional motor mistakes – can be harnessed for the production of better movement performance. Indeed, we show that improvement on the task (evidenced by increased accuracy) is accompanied by a decrease in path variability. This finding supports the explanation that variability serves the purpose of exploring possibilities in space until a suitable solution is found (the “exploration-exploitation” paradigm – see ref. 32 for a discussion). The applicability of this result in clinical settings (e.g., in physical therapy for older adults) should be explored in further experiments.

To summarize, we found that aging did not attenuate the tendency found in young individuals to increase the speed and the amplitude of their rhythmic movements in the absence of visual feedback. On the contrary, they showed a larger relative increase in those parameters compared to the young group. This increase in speed and in amplitude was maintained over repeated trials. We further found that the increase in speed and amplitude during the trial segments with no visual feedback led to improved performance which was retained during subsequent visually guided trial segments. That is, the older adults were able to adopt the more successful motor strategy, which they employed in the absence of visual feedback, and implement it when visual feedback became available.

The current results highlight the capacity of older adults to adapt movement patterns successfully after a transient perturbation. This result has implications for physical therapy in old age, and suggests that letting older adults experience a wider set of combinations of movement speeds and amplitudes (e.g., within an exercise program) can lead to more effective movement patterns and better outcomes.

## Electronic supplementary material

Supplementary Video S1

Caption for supplementary Video S1
