# Supplementary material for: Motor errors lead to enhanced performance in older adults
Source: Sci Rep. 2017 Jun 12;7:3270. doi: 10.1038/s41598-017-03430-4 (PMC5468294; doi:10.1038/s41598-017-03430-4)
Supplement: Supplementary file 2 — Caption for supplementary Video S1 [file 41598_2017_3430_MOESM2_ESM.doc]

**Supplementary Materials** for the manuscript titled **“Motor errors lead to enhanced performance in older adults”**, by S. Levy-Tzedek

**Supplementary Video S1. A demonstration of the training and the testing protocol.**

The participant is asked to perform a rhythmic movement of the forearm such that the trace of this movement on the phase plane remains within the area between the two black ellipses shown on the screen. The vertical axis denotes the speed and the horizontal one denotes amplitude.

Shown here is a demonstration of parts I and II of the training phase (static and dynamic, respectively), where the target doughnut on the screen either maintains a constant size, or is dynamically changing. These are followed by the testing phase, where the ellipse size remains constant, and the visual feedback is intermittently available. Of particular interest to note is the transition between segments B1 and V2 (marked on the video), where one can witness during the first seconds of V2 the increase in amplitude and speed of the movement which occurred during the preceding “blind” segment B1.
